# Parent–child shared book reading challenges and facilitators: a systematic review and meta synthesis

**DOI:** 10.3389/fpsyg.2025.1635956

**Published:** 2025-07-21

**Authors:** Jiaopeng Nan, Yin Tian

**Affiliations:** ^1^Institute of Education Sciences XianYang Normal University, Xianyang, China; ^2^Department of Psychology, Tianjin Normal University, Tianjin, China

**Keywords:** parents, child, shared reading, challenges, facilitators, barriers, qualitative, meta-synthesis

## Abstract

**Background:**

Parent–child shared book reading is a foundational activity that supports children’s literacy development, including language skills, cognitive growth, and social–emotional competencies. Despite its recognized benefits, various factors influence the consistency and quality of shared reading practices across different families and communities. This meta-synthesis aimed to comprehensively explore Parent–child shared book reading challenges and facilitators, drawing on qualitative research evidence.

**Methods:**

A systematic search of nine electronic databases (PubMed, Web of Science, Scopus, Medline, Embase, ScienceDirect, CINAHL, PsycINFO, and Google Scholar) was conducted in January 2025. Inclusion criteria focused on qualitative studies examining parent–child shared reading experiences among families with children under 18 years old. Studies were appraised for methodological quality using the Critical Appraisal Skills Program (CASP) checklist. Data from selected studies were extracted and analyzed using thematic synthesis, with an inductive coding process conducted through MAXQDA 24 software.

**Results:**

The analysis identified two main categories—facilitators and challenges—with ten first and 31 s categories. Facilitators included enhancing access to books and reading opportunities, providing parental support and education, creating positive and enjoyable reading experiences, ensuring high-quality book content, fostering child development through moral, social, and cognitive learning, and strengthening parental motivation. Challenges encompassed limited resources and access, sociocultural and parental barriers such as time constraints and mental health difficulties, gaps in parental knowledge and skills, and engagement challenges related to child readiness and the use of digital books. The synthesis highlighted the dynamic interplay between individual, family, and environmental factors influencing shared reading practices.

**Conclusion:**

Parent–child shared book reading is a complex, dynamic practice influenced by social, cultural, and psychological factors. Strengthening access to books, supporting parents with targeted education, and fostering enjoyable reading experiences can significantly enhance shared reading practices. Future studies should broaden cultural representation, explore digital formats, and address parental mental health to develop effective, inclusive strategies that promote early literacy and strong parent–child bonds.

## Introduction

Shared book reading involves an adult reading or sharing a book with a child, either one-on-one or in a group setting (Noble et al., 2019). The beneficial impact of shared reading on children’s language development is well-documented (Salley et al., 2022). Parent–child shared reading significantly contributes to children’s oral language development and vocabulary growth (Mak et al., 2024). Through book reading, adults introduce children to words that are not commonly used in daily conversations and clarify their meanings using the book’s content and illustrations (Mak et al., 2024). Children’s vocabulary acquisition is further enhanced when adults ask questions that encourage comprehension and highlight key vocabulary words (Wasik et al., 2016). Notably, shared reading as early as 6 months of age has been found to predict stronger vocabulary development by 12 months (O'Farrelly et al., 2018). While studied less frequently, variations in the quality of parent–child shared reading have also been shown to influence later vocabulary outcomes (Malin et al., 2014). Studies have shown that shared book reading is strongly associated with various language skills, including vocabulary development (Hindman et al., 2008), narrative abilities (Gámez et al., 2017), and phonological awareness (Lefebvre et al., 2011). Researchers have extensively examined parental involvement in preschool children’s shared book reading. Meta-analytic evidence confirms that shared reading positively influences children’s language skills (Noble et al., 2020). Compared to days without reading, incorporating shared reading into daily routines leads to significantly greater parent and child verbal interactions, as well as higher-quality language exchanges (Clemens and Kegel, 2021).

Beyond language benefits, shared reading also plays a role in fostering children’s social–emotional competence (Wirth et al., 2020). Additionally, shared reading enables parents to pass on their literacy skills and knowledge to their children (Dexter and Stacks, 2014). Shared book reading offers other significant benefits. For example, early engagement in shared reading is linked to less harsh parenting, possibly due to a reduction in disruptive behaviors associated with the activity (Jimenez et al., 2019). Moreover, shared reading serves as a valuable source of knowledge, allowing children to acquire information across various domains, including science (Venkadasalam and Ganea, 2018), mathematics (Van den Heuvel-Panhuizen et al., 2016), and moral lessons (Breitfeld et al., 2021). As a result, shared book reading plays a crucial role in expanding a child’s understanding of diverse topics (Tian et al., 2024). Shared book reading plays an important role in cognitive development, particularly by enhancing children’s memory, attention, reasoning abilities, and symbolic thinking (Shahaeian et al., 2018). Through interactive reading sessions, parents often engage children in conversations that promote the development of problem-solving abilities, contributing to broader cognitive growth. These shared experiences expose children to valuable learning activities, such as interpreting text meaning, discussing abstract ideas, and drawing logical connections within a narrative (Hindman et al., 2014). Additionally, reading picture books encourages children to tackle problem-solving challenges, express their perspectives, ask questions, and explore various answers—skills that support both general learning and academic success (van Bergen et al., 2017).

Findings from international large-scale assessments also suggest that early shared book reading is linked to later reading achievement (Mullis et al., 2012). Additionally, shared reading contributes to academic success, showing positive associations with pre-schoolers’ academic skills (Mann et al., 2021) and school readiness (Anthony et al., 2014). Due to its profound impact, shared reading inspiring the creation of interventions aimed at enhancing shared reading practices (Salley et al., 2022).

As previously stated, shared book reading is a dynamic and interactive practice that shapes children’s language development, cognitive growth, and social–emotional well-being. While quantitative studies have established the benefits of SBR, they often fall short of capturing the nuanced, real-life contexts in which reading practices occur. In contrast, qualitative research offers valuable insights into how parents experience and navigate shared reading, allowing for an in-depth exploration of cultural, emotional, and logistical factors that influence engagement.

A qualitative approach allows researchers to explore how parents navigate shared reading practices, the challenges they encounter, and the factors that influence engagement. By examining parental narratives, observational data, and interactive reading behaviors, researchers can uncover the underlying mechanisms that contribute to effective shared reading experiences. These insights can inform the development of tailored interventions, such as culturally responsive reading materials, training programs for parents, and strategies that encourage meaningful dialogue between caregivers and children.

Despite strong evidence promoting shared reading, significant disparities persist in how consistently and meaningfully families engage in it. Understanding the facilitators that promote, and the barriers that hinder, parent–child shared book reading is critical to closing this gap. These factors play a pivotal role in shaping early literacy environments, particularly among socioeconomically or culturally diverse populations.

Therefore, the objective of this meta-synthesis was to systematically synthesize qualitative research to identify the key facilitators and challenges that influence parent–child shared book reading practices, with the aim of informing effective, inclusive strategies to support early literacy development.

## Methods

Meta-synthesis is a qualitative research method used to analyze multiple qualitative studies identified through a systematic review. This approach can be applied to multiple studies conducted by a single author on a specific topic or to the findings of studies conducted by various researchers within a particular field (Sandelowski et al., 1997). The review adhered to the ENTREQ guidelines (Tong et al., 2012) which provide a standardized framework for conducting qualitative syntheses. The qualitative synthesis was grounded in critical realism (Bhaskar, 2013) as its theoretical foundation. According to critical realism, reality consists of multiple layers: the actual world, which exists independently of our observation; the underlying mechanisms and processes shaping this reality; and the subjective experience of reality as perceived through our sensory processing. This approach is particularly relevant to exploring parent–child shared book reading, as it recognizes both the observable behaviors—such as reading frequency, book choice, and interaction styles—and the underlying mechanisms that influence them, including parental beliefs, emotional bonds, cultural norms, and structural conditions (e.g., time, space, resources).

This study analyzed the findings of published qualitative research carried out by researchers on the topic of Parent–child shared book reading.

### Inclusion criteria

The studies included in our review met the following criteria:

Population: Children under the age of 18 (Lansdown and Vaghri, 2022), biological parents (mothers, fathers), legal guardians, adoptive parents, or caregivers who act in a parental role. No restrictions were placed on the developmental status of the children. Studies were included regardless of whether the children were typically developing or had disabilities, as long as the focus was on shared book reading. However, none of the included studies specifically targeted children with developmental disabilities.

Intervention: Parent–child shared book reading experiences.

Comparison: Not applicable (qualitative studies typically do not have direct comparisons).

Outcome: Experiences, perceptions, challenges, and facilitators related to parent–child shared book reading, specifically from the perspective of parents, legal guardians, adoptive parents, or caregivers acting in a parental role. Studies focused on teachers, children, or other professionals were excluded, as the objective of this synthesis was to explore shared reading within the home and family context.

Study design: Qualitative studies using interviews, focus groups, phenomenology, grounded theory, content analysis, thematic analysis etc. Mixed-methods studies with a qualitative component relevant to the research question.

Other criteria: Published in peer-reviewed journals, written in English and no time restrictions.

### Exclusion criteria

Studies were excluded if they were Observational or secondary data studies, study focuses on the effect of an intervention, quantitative studies, literature reviews, theoretical or position papers, editorials, conference papers, and non-peer-reviewed articles, lacking clear descriptions of methodological details, of inadequate methodological quality, published in non-English language. Notably, quantitative studies and those focused solely on the measurable effectiveness of interventions were excluded. However, qualitative studies conducted in the context of an intervention were included if they explored the experiences, perceptions, or practical implementation of shared book reading strategies from the perspective of parents. Our focus was on the qualitative depth of engagement, rather than on intervention outcomes.

The search was limited to only peer-reviewed articles and because we believed conceptual saturation to be reached based on those articles, we opted not to conduct further searches of “gray” literature (Thomas and Harden, 2008).

### Search strategy and screening

Nine electronic databases were searched (PubMed, Web of Science, Scopus, Medline, Embase, ScienceDirect, CINAHL, PsycINFO and Google Scholar). Since Google Scholar is a search engine and may provide a lot of scattered data, we did not use the search strategy we used for other databases and only the most relevant articles were selected. Qualitative research studies were targeted, and mixed-method studies were also screened to determine whether the qualitative component met the inclusion criteria for this review. In order to achieve the best search strategy, a combination of the following keywords and MeSH terms was used: (“Shared book reading” OR “Shared novel reading” OR “Shared text reading” OR “Joint reading” OR “Interactive reading”) AND (“challenges” OR “Barries” OR “facilitators”) AND (“qualitative” OR “interview” OR “thematic analysis” OR “phenomenology” OR “grounded theory” OR “focus group” OR “content analysis”). The searches were conducted on Jan 2025. A total of 4,440 articles were identified using this search strategy, which were uploaded into Endnote to facilitate the screening process. The number of papers reduced to 1856 when duplicates were removed.

**Figure 1:**
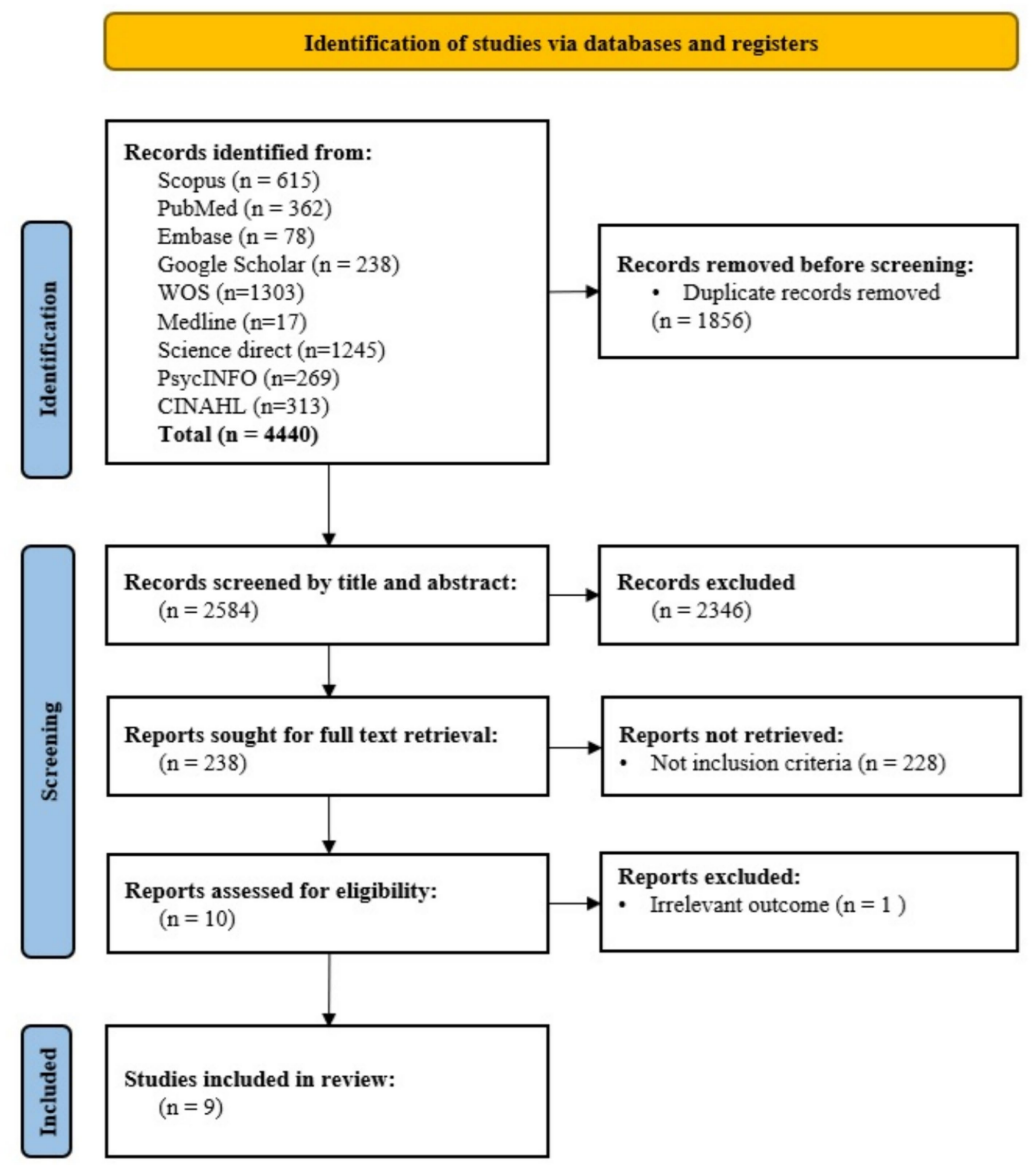
Flowchart of screening process.

The researchers screened studies for inclusion in a meta-synthesis. They used the title and abstract to assess whether papers met the criteria. Two researchers independently screened a sample of studies, and there was moderate agreement between them. Disagreements were resolved through discussion. A total of 238 articles were selected for full-text screening, and there was near-perfect agreement between the two researchers at this stage. A total of nine articles met the inclusion criteria and were included in the meta-synthesis. See Figure 1 for the PRISMA flowchart.

### Data extraction

Data extraction was conducted independently by two reviewers using a structured template developed in accordance with the thematic synthesis approach outlined by Thomas and Harden (2008) and guided by ENTREQ (Enhancing Transparency in Reporting the Synthesis of Qualitative Research) recommendations. For each included study, we extracted the following information: author(s), year of publication, and country; study title and qualitative design (e.g., phenomenology, content analysis, case study, co-design, or mixed methods); participant details and sample size, specifically focusing on parents, caregivers, or guardians acting in a parental role; and format of data collection, such as whether semi-structured interviews, focus groups, telephone interviews, or observations were used. We also recorded the analytical approach employed by each study, including methods like thematic analysis, inductive content analysis, or other coding procedures. The “Results,” “Findings,” or “Conclusions” sections of each article were reviewed in depth, and relevant thematic narratives and participant quotes related to shared book reading practices were extracted (see Table 1). The initial data extraction was carried out using Excel, and qualitative findings were subsequently imported into MAXQDA 20 for line-by-line coding. Any discrepancies between the reviewers were discussed and resolved through consensus.

**Table 1:** Information extracted from the articles reviewed in the meta-synthesis.

Country (References)	Title	Type of study	Participants/number	Format for data collection	The data analysis	Results	Quality
Queensland (State of Australia) (Smith et al., 2023)	Co-designing a shared book reading environment at a community hub	Co-design	Father, mother or grandmother/12	Semi-structured interviews Focus groups and priority setting	Inductive content analysis	1- Increase opportunities for shared book reading at the hub 2- Give families more information about how to share books with their children 3- Demonstrate how books can be shared with children	High = 17
United States (Boit et al., 2024)	Learning Together Through Shared Book Reading: Experiences of Burmese Refugee Mothers and Their Pre-schoolers	Qualitative case study	Mothers/4	Interviewing and observations	Coding qualitative data	1- Language Dynamics Within Communication 2- Comfort and Engagement	High = 17
Norway (Kucirkova and Grøver, 2022)	The Importance of Embodiment and Agency in Parents’ Positive Attitudes Towards Shared Reading with Their Children	Qualitative study	Parents/24	Telephone interviews	Thematic analysis	1- The importance of children being actively engaged and interested in the reading activity 2- Controversies surrounding the use of digital books	Moderate = 15
Southeast Queensland, Australia (Dunstan et al., 2024)	Exploring the impact of a co-designed shared book reading environment for families in a community hub	Co-design	Parents/carers/46	Inductive content analysis	Inductive content analysis	1- Increasing access to a range of books 2- Increasing awareness of book reading 3- Using reading center staff to facilitate the pleasure of reading 4- Increasing engagement with book borrowing 5- Changes in reading behaviors at home	High = 18
Israeli (Deitcher et al., 2019)	Book selection for shared reading: Parents’ considerations and researchers’ views	Qualitative study	Parents/104	Semi-structured interviews	Coding qualitative data	1- Reading for pleasure 2- book’s linguistic level 3- Rhythm, rhyme, and flow of book 4- Opportunity to impart morals 5- Social–emotional support 6- Enriching knowledge 7- Text length of book 8- Importance of a book’s appearance 9- Importance of a book’s structure	Moderate = 15
South African (Coetzee et al., 2023)	A shared reading intervention: Changing perceptions of caregivers in a semi-rural township	Qualitative study	Caregivers (mother, father, grandparents, sibling, uncle)/40	Semi-structured interview	Thematic analysis	1- Absence of a culture of book sharing 2- Reading for education rather than recreation 3- Lack of access to books and reading material and perceived limited book-sharing skills 4- Limited time	High = 17
England (Swain et al., 2017)	‘We occasionally miss a bath but we never miss stories’: Fathers reading to their young children in the home setting	Mixed methods	Fathers/18	Semi-structured interviews	Inductive content analysis	1- Enjoyment of Reading 2- Reproduction of Childhood Practices 3- Bonding and Building Intimacy 4- Sense of Responsibility 5- Planning and Structuring Reading Times 6- Supporting Child Choice and Interest 7- Fathers’ busyness and fatigue after work 8- Lack of adequate space at home 9- Lack of awareness in choosing the type of book 10- Unsure of how to read a book	Moderate = 14
United states (Crosh et al., 2024)	Beliefs and Motivations Regarding Early Shared Reading of Parents from Low-Income Households	Qualitative study	Parents/21	Semi-structured interviews	Inductive approach	1- competing demands on time 2- lack of resources 3- parental mental health 4- it’s too early/the baby is not ready 5- parents as child’s first teachers 6- reading catalyzes the child’s development. 7- reading begets more reading (positive feedback loop) 8- bonding with the infant 9- it works 10- “two-for-one” shared reading	High = 17
United Kingdom (Preece and Levy, 2020)	Understanding the barriers and motivations to shared reading with young children: The role of enjoyment and feedback	Mixed methods	Parents/20	In-depth interviews	Open coding and axial coding	1- Children’s enjoyment 2- Parental enjoyment through positive feedback 3- Child’s lack of enjoyment is a barrier to shared reading	High = 16

### Quality assessment

To make sure the research articles were unbiased, two reviewers assessed them using a checklist called CASP (CASP^1^). CASP is a set of 10 guidelines that researchers often use to evaluate how well qualitative research is designed and reported. These guidelines cover areas like the research goals, the methods used, and how the data was analyzed. Researchers can use different qualitative methods depending on their beliefs about how knowledge is gained (epistemology). Because of this, the reviewers focused on whether the authors considered each of the CASP factors, rather than having a one-size-fits-all set of rules. Two researchers reviewed each article using CASP, and they talked about any disagreements they had. Although CASP does not employ a formal scoring system, studies are typically categorized as high, moderate, or low quality based on their adherence to the checklist criteria. A widely used scoring approach assigns 2 points for “yes” (criterion met), 1 point for “cannot say” (partially met or unclear), and 0 points for “no” (not met), yielding a total score ranging from 0 to 20. Accordingly, studies scoring between 16 and 20 are classified as high quality, those scoring between 10 and 15 as moderate quality, and those below 10 as low quality (CASP, 2018). In the end, all the articles were included in the analysis, even if they had some weaknesses, because they all provided valuable information.

### Synthesis

The thematic synthesis followed the process outlined by Thomas and Harden (2008), using an inductive approach with MAXQDA 24 software. In the first stage, each unit of meaning from the extracted data was coded line by line, facilitating the translation of concepts across the included studies. After coding was completed, all codes were reviewed to incorporate any necessary additions or to merge similar ones. The second stage involved forming descriptive sub categories by organizing codes hierarchically, with new codes created to capture the shared meanings within these groups. In the third stage, analytic main categories were developed, extending beyond the findings of the original studies. At this stage, the review questions became central, guiding the synthesis of descriptive categories to generate new insights. Quotes were selected to illustrate the categories clearly and to reflect a broad range of studies included. A summary of the analysis is provided in Table 2.

**Table 2:** Overview of main first and second categories.

Main categories	First-categories	Second-categories
Facilitators	Enhancing access and opportunities	- Increasing access to a range of books - Improving opportunities for shared book reading - Promoting engagement with book borrowing
	Parental support and education	- Providing parents with strategies and information - Increasing awareness about reading benefits
	Creating positive reading experiences	- Encouraging reading for pleasure - Fostering children’s active engagement - Planning and structuring reading times - Reproducing positive childhood reading practices - Strengthening parent–child bonding and intimacy
	Book and content quality	- Selecting books with appropriate language level - Rhythm, rhyme, and flow enhancing engagement - Suitable text length, attractive appearance, and structure
	Child development	- Imparting morals through stories - Promoting social–emotional growth - Enriching children’s knowledge
	Parental motivation	- Sense of responsibility toward supporting children’s reading - Child-parents enjoyment
Challenges	Limited resources and access	- Lack of access to books and reading spaces - Lack of adequate space at home
	Sociocultural and parental barriers	- Absence of a culture of book sharing - Parental Busyness, Fatigue, and Limited Time for Book Sharing - Parental mental health
	Knowledge and skill deficits	- Lack of awareness in choosing appropriate books - Unsure of how to share books effectively - Limited book-sharing skills
	Child engagement and communication challenges	- Controversies around the use of digital books - Age

## Results

The information extracted from the selected articles and their findings is provided in Table 1. Methodological approaches used in these 9 articles included Co-design (*N* = 2), qualitative content analysis (*N* = 5), and mixed methods (*N* = 2). Studies were performed in the USA (*N* = 2), Australia (*N* = 2), the UK (*N* = 2), Norway (*N* = 1), Israeli (*N* = 1), and South African (*N* = 1). Most participants in the studies were parents (*N* = 7), while one study included only mothers and another included only fathers. Sample sizes were between 4 and 104 participants.

Two main categories were extracted: facilitators and challenges. Each main category is structured around identified first and second categories, with thorough explanations and supporting quotes. The following are the two categories and their first and second categories:

### Facilitators of Parent–child shared book reading

#### Enhancing access and opportunities

##### Increasing access to a range of books

Access to diverse and appropriate reading materials is crucial for fostering a culture of literacy among children. One article, illustrate how increasing the variety of books available in community hubs led to higher engagement levels (Dunstan et al., 2024). Participants noted that improved access allowed children to explore different genres and topics, enhancing their interest in reading. One parent emphasized the importance of this access by stating, “The selection of books here is amazing; my child cannot go wrong in choosing any of them. It’s exciting to see him pick something new every time we come (Dunstan et al., 2024).” This highlights how varied options spark curiosity and encourage children to develop a love for reading, reinforcing the idea that exposure to multiple genres is essential for nurturing young readers. Importantly, families were not only engaging with books on-site but were also borrowing them for use at home, thereby enhancing exposure to reading beyond the structured setting. This improved access supported greater parental involvement and offered children a chance to explore topics of interest within their own homes, reinforcing sustained engagement with books.

##### Improving opportunities for shared book reading

Organizing structured activities at community hubs significantly boosts opportunities for shared reading. One article highlights how community events, such as themed reading days, increased the frequency of reading sessions (Smith et al., 2023). This structured approach allows families to engage collectively in reading, providing them with social support and shared experiences that enrich the reading practice. While these events were initially community-based, but they contributed to parents adopting more intentional shared reading practices at home. A participant remarked, *“Now, during these events, I see more families gathered around books, laughing and sharing stories. It has really brought our community together* (Smith et al., 2023).*”* This underscores the role of community initiatives in creating an environment that promotes reading as a communal activity, facilitating connections among families and reinforcing the social aspect of reading. Thus, community-based opportunities served as models that enriched the home literacy environment by providing both the confidence and tools to continue shared reading independently.

### Promoting engagement with book borrowing

Awareness of borrowing processes at community hubs has been linked to improved engagement in reading practices. One article notes that when families are informed about borrowing options, they are more likely to participate in reading activities (Dunstan et al., 2024). This engagement fosters a sense of responsibility in children, motivating them to take ownership of their reading habits. As one participant stated, *“I did not know we could borrow books until recently, and now my kids are excited to pick their favorites to take home. It feels great that they want to read more* (Dunstan et al., 2024).*”* This illustrates how clear communication about borrowing procedures can enhance children’s proactive involvement in their reading journey, making them feel more connected to the reading community. However, this transfer of reading materials from the community to the home space contributed to a richer home literacy environment, where reading became a more consistent and child-led activity.

#### Parental support and education

##### Providing parents with strategies and information

Equipping parents with effective strategies for engaging in shared reading is vital. Demonstrating Effective Book-Sharing Practices Workshops and demonstrations that model effective reading techniques enhance parents’ skills. One article emphasizes the need for educational resources that outline practical book-sharing strategies (Smith et al., 2023). When parents receive this support, they feel more confident in their ability to foster literacy development, which in turn encourages more frequent shared reading. A mother expressed her need for guidance, stating, *“We do not have the knowledge or skills to know what books are best for our kids. Workshops made me realize I can actually help them learn and enjoy reading* (Smith et al., 2023).*”* Or somewhere else, participants appreciated the workshops, with one father noting, *“Attending the workshop opened my eyes to how I can make reading fun. I used to just read the words, but now I know I can interact more* (Smith et al., 2023).*”*

The specific strategies and practices encompassed a range of interactive and responsive book-sharing techniques. These included dialogic reading methods (e.g., asking open-ended questions, prompting children to predict or recall story elements), encouraging child-led book selection, using expressive voice and gestures, incorporating storytelling into daily routines (such as bedtime), and engaging in shared reflections during or after reading. Parents also learned to pause during reading to invite comments, relate the story to the child’s own experiences, and support vocabulary development by explaining unfamiliar words. Workshops and demonstrations provided parents with hands-on examples of how to implement these techniques, empowering them to transform reading sessions from passive to participatory experiences.

##### Increasing awareness about reading benefits

Raising awareness of the cognitive and emotional benefits of reading is essential for changing perceptions around shared reading. One article illustrates how understanding the positive impacts of reading on child development can motivate parents to prioritize reading activities, leading to increased engagement (Dunstan et al., 2024). One parent remarked, *“I never realized how much reading together could help my child’s vocabulary and confidence. Now, I make it a point to read daily* (Dunstan et al., 2024).*”* This indicates that when parents recognize the value of reading, they are more likely to integrate it into their daily routines, thus enhancing their children’s literacy development.

#### Creating positive reading experiences

##### Encouraging reading for pleasure

Fostering a love of reading hinges on encouraging enjoyment rather than viewing reading solely as an educational task. Articles discuss how parents who emphasize pleasure in reading report more positive interactions with their children, leading to increased engagement and enthusiasm (Deitcher et al., 2019; Swain et al., 2017). A participant noted, *“For us, reading is about enjoyment, not just lessons. We love to pick funny stories that make us all laugh* (Deitcher et al., 2019).*”* This highlights that prioritizing enjoyment in reading can transform the experience, making it a cherished family activity that builds bonds and fosters a lifelong love for books.

##### Fostering children’s active engagement

Allowing children to choose their reading materials promotes autonomy and interest in reading. One article highlights that children who select their books show greater enthusiasm and are more likely to participate actively during reading sessions (Swain et al., 2017). One mother reported: *“He [her 1.5-year-old son] is probably more there that he likes to just hold them [the books] himself and browse through them. And if mom does it with him, then he will have some quiet time in a way. Because the books are very large right. And because he has, the interest, in that they are big and colorful, so he would like to do it a little himself as well.”* Another mother of a two-and-half-year-old son shared that when they read a book, the boy was actively contributing before and during the SBR session: *“Then he sits on my lap and then he brings a book… And he likes to flip in the books too. And he then flips through the book completely incoherently, that is to say… but since there are not much plot-based stories, to a large extent, it has not played that much of a role. But he is active, it is how he is like… how is like when he reads, he is active, yes* (Swain et al., 2017).*”* This emphasizes the importance of autonomy in fostering a child’s intrinsic motivation to read, which can lead to more regular reading habits and a stronger connection to literature.

##### Planning and structuring reading times

Establishing a routine around reading times enhances the experience for both parents and children. One study indicates that structured reading times, such as bedtime stories, create intimate moments that strengthen the bond between parent and child, contributing to a positive reading environment (Swain et al., 2017). One mother described, *“Every night, we have our reading time before bed. It’s our special moment, and it calms her down and helps her sleep* (Swain et al., 2017).*”* This illustrates how consistent reading routines can provide comfort and security for children, making reading a significant part of their daily lives.

##### Reproducing positive childhood reading practices

Many parents draw from their own positive reading experiences from childhood to inform their practices with their children. One article illustrates how recollections of being read to inspire current reading habits, creating a continuity of positive reading experiences across generations (Swain et al., 2017). A father reflected, *“My dad always read to me, and I want to recreate that coziness with my kids. It’s a tradition I cherish* (Swain et al., 2017).*”* This emphasizes the impact of intergenerational reading practices, where positive experiences influence current behaviors and attitudes toward reading.

##### Strengthening parent–child bonding and intimacy

Shared book reading serves as a powerful tool for strengthening the bond between parents and children, enhancing emotional intimacy and fostering a sense of connection. Engaging in reading together creates a unique opportunity for parents and children to share experiences, emotions, and ideas in a safe and nurturing environment. This theme is prevalent throughout the findings, particularly highlighted in one article, where parents discuss the intimate moments that arise during shared reading sessions (Swain et al., 2017).

When parents read aloud to their children, they are not merely transferring information; they are also creating a shared experience that can deepen their relationship. A mother expressed, “*Every night, we have our reading time before bed. It’s our special moment, and it calms her down and helps her sleep* (Swain et al., 2017).” This quote underscores the importance of establishing routines around reading, which not only provide comfort but also serve as a dedicated time for connection. The physical closeness that comes from snuggling up with a book allows for affectionate gestures, such as hugs and cuddles, which further enhance feelings of safety and love.

#### Book and content quality

##### Selecting books with appropriate language level

The language level of books emerged as a critical factor in parental selection during shared reading. Parents consistently favored books that offered a balance between introducing new vocabulary and maintaining overall linguistic accessibility. In addition to vocabulary, they valued texts with developmentally appropriate grammar, clear and concise sentence structures, and coherent narrative flow suited to the child’s cognitive and linguistic stage. The included studies highlight the importance of selecting books that are not only understandable and engaging but also sufficiently rich to support language development without causing frustration or disengagement (Deitcher et al., 2019; Boit et al., 2024; Pramling Samuelsson et al., 2025). A parent stated, *“I look for books that introduce new words but are still understandable. It’s important for my child to learn without getting frustrated* (Deitcher et al., 2019).*”* This highlights the balance parents seek in selecting literature that is both engaging and educational, ensuring that children are motivated to read while developing their language skills.

##### Rhythm, rhyme, and flow enhancing engagement

Books that incorporate rhythm and rhyme significantly enhance engagement. One article notes that parents find these elements appealing, as they make reading enjoyable and encourage children to participate actively in the reading process (Deitcher et al., 2019). A participant commented, *“When I read rhyming books, my child loves to join in. It feels like a game, and she gets excited about the stories* (Deitcher et al., 2019).*”* This illustrates how rhythmic and playful language can transform reading into a fun and interactive experience, further encouraging children to engage with texts.

##### Suitable text length, attractive appearance, and structure

In addition to content, parents emphasized the importance of a book’s physical and structural characteristics when selecting materials for shared reading. Studies reported that text length was a practical consideration, with parents preferring shorter or moderately long books that could be completed in one sitting without overwhelming the child or disrupting daily routines (Deitcher et al., 2019). Books that were too lengthy often led to loss of attention or unfinished sessions, which some caregivers viewed as diminishing the impact of the reading experience. Furthermore, parents favored books with visually engaging illustrations, clear layouts, and simple, predictable structures that supported children’s understanding and sustained interest. One parent noted, *“Books with colorful pictures and simple text keep my child’s attention. She loves pointing out what she sees* (Deitcher et al., 2019).*”* These findings underscore that appropriate text length, combined with an attractive visual design and clear narrative structure, significantly influences children’s engagement and enhances the effectiveness of shared book reading.

#### Child development

##### Imparting morals through stories

Shared reading serves as a medium for teaching morals and values. One article discusses how parents often select books that provide opportunities for discussing important life lessons, using literature as a tool for moral education (Deitcher et al., 2019). A mother noted, *“I want my child to learn important values through stories. It helps us talk about right and wrong in a fun way* (Deitcher et al., 2019).*”* This demonstrates how literature can be leveraged to instill ethical lessons, making reading not just an educational activity but also a means of imparting life skills.

##### Promoting social–emotional growth

Reading together supports emotional development and helps children navigate social situations. This theme is particularly emphasized in one article (Deitcher et al., 2019), which discusses how reading can address social–emotional concerns through relatable narratives that resonate with children’s experiences. A participant expressed, *“Reading stories about friendship helps my child understand how to make friends and deal with emotions* (Deitcher et al., 2019).*”* This indicates that literature can serve as a valuable tool for social–emotional learning, allowing children to explore and understand their feelings and relationships in a safe context.

##### Enriching children’s knowledge

Reading is viewed to expand a child’s knowledge base. Parents in articles express that good books not only entertain but also educate, providing information alongside storytelling that enriches children’s understanding of the world (Deitcher et al., 2019; Crosh et al., 2024). One parent shared, *“Books open up new worlds for my child. She learns about animals, places, and ideas that she would not encounter otherwise* (Deitcher et al., 2019).*”* This reflects the multifaceted role of reading in cognitive development, where exposure to diverse topics through literature enhances children’s knowledge and curiosity.

#### Parental motivation

##### Sense of responsibility toward supporting children’s reading

Many parents feel a strong sense of duty to engage in reading with their children, viewing it as part of their role. One article highlights how this sense of responsibility drives parents to prioritize reading time, even amidst busy schedules (Swain et al., 2017). A father remarked, *“Reading is something I must do for my kids. It’s my responsibility to help them learn and grow* (Swain et al., 2017).*”* This underscores the deep commitment parents have towards fostering their children’s literacy, viewing it as an essential aspect of their parenting.

##### Child–parent enjoyment

The mutual enjoyment derived from reading sessions motivates parents to continue these practices. As seen in one article (Swain et al., 2017), parents report that positive interactions during reading enhance their commitment to regular reading activities, creating a cycle of reinforcement. One mother stated, “*I do enjoy it. It’s a bit of time at the end of the day just for us, and it’s something my child looks forward to* (Swain et al., 2017).”

Furthermore, when children see their parents enjoying the reading process, it cultivates a love for books and learning. Positive interactions during reading can lead to increased enthusiasm for stories, making children more likely to seek out books independently. As noted in one article, “*When parents show excitement about reading, children mirror that enthusiasm, leading to a more enriching experience* (Preece and Levy, 2020).” This mirrors the idea that enjoyment is contagious; when parents express joy in reading, it encourages children to embrace the activity with similar enthusiasm.

### Challenges of parent–child shared book reading

#### Limited resources and access

##### Lack of access to books and reading materials

A significant barrier to reading is the limited access to books and reading materials in many households. Articles discuss how socioeconomic factors restrict families’ abilities to acquire books, hindering shared reading practices (Crosh et al., 2024; Coetzee et al., 2023). Many families reported having very few books, with one participant stating, *“I do not have children’s books. They are too expensive, and I cannot afford them* (Coetzee et al., 2023).*”* This highlights a critical gap in resources that directly impacts children’s literacy opportunities and emphasizes the need for community support in providing accessible reading materials.

##### Lack of adequate space at home

Physical space for reading can also be a challenge. Parents in one article noted that inadequate space in their homes makes it difficult to create a conducive reading environment, further limiting the opportunities for shared reading experiences (Swain et al., 2017). This indicates that logistical challenges, such as space and energy levels, can hinder the establishment of regular reading habits, necessitating solutions that accommodate families’ living situations.

#### Sociocultural and parental barriers

##### Absence of a culture of book sharing

Across the included studies, participants frequently reported that shared book reading was not a normative or culturally embedded practice in many households. One key reason cited was the perception of reading as an educational task rather than a recreational or bonding activity. This framing limited parental engagement with reading outside of school-related purposes. As one mother explained, *“I think that this is something that comes in older years, like grade 1. We do not really read at home* (Coetzee et al., 2023),*”* illustrating how reading was viewed as appropriate only in formal educational contexts.

This perception was reinforced by cultural experiences in which shared reading was absent from participants’ own childhoods. One participant noted, *“I have no memories of anyone sharing books with me as a child. It just wasn’t part of my upbringing”* while another, acting in the role of a sister, reflected, *“Reading with her when she is so small honestly never crossed my mind* (Coetzee et al., 2023).*”* These accounts highlight the intergenerational and cultural barriers that contribute to the lack of a book-sharing tradition in many families.

##### Parental busyness, fatigue, and limited time for book sharing

The demands of modern family life often limit the time and energy parents can devote to shared book reading. Both busyness and fatigue emerged as significant barriers, particularly for working parents managing multiple responsibilities. One study emphasized that fatigue after long workdays frequently reduces the frequency and quality of shared reading, even when parents are motivated to engage. One participant shared, “*We have busy lives and I get home from work and sometimes we have tea together and sometimes we do not so 7.30 bedtime is when I have a chance to review the day and have a chat about what was done and I would not get away without it because they are mad keen on books themselves. I do not have a choice!* (Swain et al., 2017)*.”*

In addition to physical exhaustion, limited available time due to work, household obligations, and children’s extracurricular activities often constrains the opportunity for book sharing. This time scarcity can lead to missed moments of parent–child connection and reduce the consistency of literacy routines at home. As two mothers remarked, “*I come home late from work. I work for long hours and then I am too tired when I come home* (Coetzee et al., 2023).”

These findings underscore the need for flexible and realistic strategies that help families integrate shared reading into daily life, even in short or informal formats.

##### Parental mental health

Parental mental health plays a crucial role in the dynamics of shared book reading, significantly influencing both the frequency and quality of these interactions. When parents experience mental health challenges, such as stress, anxiety, or depression, their ability to engage in nurturing and supportive activities with their children can be severely impacted. One article emphasizes that mental well-being directly affects parents’ energy levels, emotional availability, and overall engagement in their children’s lives, including reading practices (Crosh et al., 2024).

Parents struggling with their mental health may find it difficult to prioritize activities like reading, which require focus and emotional connection. When discussing things, they had experienced that affected their ability to read to their baby, several mothers cited poor mental health. One mother said that: *“her depression was so profound that her baby, “wasn’t even on my map at times.” Then when asked what she would share with other mothers about shared reading, she said that reading to a child might help you feel better. When asked about general barriers, a mother from the reading group shared how profoundly she struggled after the death of her partner: “Personally, if I had to say that I’m struggling with anything, it’s just the loss of my kids’ father…* (Crosh et al., 2024).*”*

This highlights a common barrier where mental health challenges can lead to a withdrawal from interactive and bonding activities, causing both parents and children to miss out on the benefits of shared reading.

Moreover, the stressors associated with mental health issues can create a cycle of negative emotions that further hinder reading interactions. Parents may feel guilt or inadequacy for not engaging in reading as often as they would like, which can exacerbate feelings of anxiety or depression. This emotional strain can lead to a lack of enjoyment in reading, transforming it from a joyful experience into yet another source of pressure.

#### Knowledge and skill deficits

##### Lack of awareness in choosing appropriate books

Parents often express uncertainty about how to select age-appropriate or engaging books for their children. One article highlights the need for guidance in book selection to foster effective reading practices and enhance children’s literacy development (Swain et al., 2017). This indicates a gap in knowledge that can limit children’s engagement, emphasizing the necessity for resources that guide parents in making informed choices.

##### Unsure of how to share books effectively

Many caregivers lack confidence in their reading techniques, which can hinder their engagement in shared reading. As noted in one article, providing training and resources can help parents develop effective reading strategies that promote better interactions with their children (Swain et al., 2017). This illustrates the potential benefits of instructional resources that can enhance parents’ skills and make shared reading a more interactive experience.

##### Limited book-sharing skills

The belief that they lack the necessary skills for effective book sharing can deter parents from engaging in reading activities. As noted by a 17-year-old sister: *“I have no idea how to do that [share books]* (Coetzee et al., 2023).*”* One article suggests that targeted support and education can alleviate these concerns, encouraging more families to participate in shared reading practices (Coetzee et al., 2023). This highlights the importance of empowering parents with the skills and confidence needed to facilitate meaningful reading experiences for their children.

#### Child engagement and communication challenges

##### Controversies around the use of digital books

In addition to preferences for content, language, and physical presentation, the included studies revealed mixed parental attitudes toward digital books. While some parents acknowledged the convenience of digital formats—especially for families with limited physical access to books—others expressed concern about screen time, distraction, and reduced interactivity during shared reading. One study noted that parents were often hesitant to use digital books with younger children, fearing that screens would interfere with attention spans or lead to passive consumption rather than active engagement. One participant in one article reported: “*Yes then, we did read digital books, but it was such a struggle. He [the mother’s son] was more concerned with getting the tablet wasn’t he? You know when I read on my mobile or iPad, he is very curious about it and wants it himself then (…) Yes, so that’s what’s so dangerous about technology, that children want to control and own them. I’m trying to keep him away from technology* (Kucirkova and Grøver, 2022).” Conversely, a smaller number of participants reported that digital books, when designed with interactive features and narration, could support emergent literacy and maintain children’s interest. These conflicting views reflect an ongoing tension between accessibility and quality of engagement in digital versus print media. The findings suggest a need for clearer guidance on how and when digital books can be effectively integrated into shared reading routines without compromising developmental benefits.

##### Age

In one article, parents often express concerns that their children may not be ready for shared reading activities, particularly with younger infants or toddlers. One parent articulated this sentiment by saying, *“…as of right now, she’s just not into it.*”

Although many of them recognized the importance of reading, they questioned the timing of introduction. Some interpreted their infant falling asleep while reading as evidence that they were not ready, *“…baby is not ready for this yet.”* Some also interpreted infant interactions with the book as signs they were not ready, *“.so [Dad] was just reading aloud to try to, he was like, she wasn’t interested. She wanted to rip my pages or do that, just make noise.”* One parent noted that they were not accustomed to the notion of reading to a baby at this young age, and therefore had not started shared reading, *“I never seen nobody read to a little bitty-bitty baby like that before* (Crosh et al., 2024).*”*

This highlights a common hesitation among parents regarding the appropriateness of introducing reading at an early age, reflecting a belief that infants may not yet be prepared for the complexities of shared reading experiences.

## Discussion

This review synthesized evidence from nine qualitative studies to explore the facilitators and challenges of parent–child shared book reading. The results offer a nuanced understanding of the social, environmental, psychological, and developmental factors that shape this practice. Several main categories were identified as facilitators: access and opportunities for reading, parental support and education, creation of positive reading experiences, quality of books and content, developmental benefits for children, and parental motivation. These findings are discussed in detail below, supported by relevant literature.

### Enhancing access and opportunities

Access to books and reading environments emerged as a foundational facilitator. When families had access to diverse and culturally relevant reading materials—either through community hubs, libraries, or home libraries—they were more likely to engage in regular shared reading. Neuman and Celano found that children from print-rich environments had significantly more exposure to books and literacy-related interactions compared to those from print-poor neighbourhoods (Neuman and Celano, 2001). Similarly, a systematic review by Mol et al. showed that shared reading interventions that provided books were associated with greater language and literacy gains (Mol et al., 2008). Pretorius highlights that access to age-appropriate books is essential for fostering a reading culture. Children need both access to books and the presence of a capable role model, such as a parent or older sibling, to help instill a love for reading (Pretorius, 2014).

The availability of structured opportunities—such as book borrowing programs and story time sessions—was another critical factor. These settings not only facilitated access to books but also promoted a sense of community engagement and modelled reading behaviors.

Participant responses clearly indicated that the rise in book borrowing was linked to increased awareness that books were available for loan from the hub, along with project-based incentives such as book borrowing cards and stickers (Dunstan et al., 2024). This heightened borrowing activity holds significant meaning for families in this study, particularly as many have limited access to books at home due to the effects of social determinants of health and socioeconomic disadvantage (Manz et al., 2010; Pace et al., 2017). For these vulnerable families, finding alternative methods to access books is crucial. Future research might explore the role of digital tools—such as e-readers and e-books—alongside traditional print books to further expand access. Engagement with public libraries has also been shown to encourage parents to read aloud to their children at home (Chen et al., 2016). However, families in under-resourced areas often face barriers to using libraries, including limited awareness, lack of familiarity, or undervaluing of library services (Campana et al., 2022). A longitudinal study by Barratt-Pugh and Allen (2011) on the Better Beginnings program in Western Australia demonstrated that distributing books as part of a literacy initiative increased both the frequency of reading and children’s engagement with books.

### Parental support and education

Parental knowledge and confidence in using effective shared reading strategies were repeatedly emphasized as key enablers. When parents were taught techniques such as dialogic reading, questioning, and engaging the child through voice modulation or gestures, they reported higher confidence and engagement levels. Whitehurst et al. introduced dialogic reading as an evidence-based practice that transforms reading into an interactive dialogue rather than a passive activity, significantly boosting children’s expressive language development (Whitehurst et al., 1988).

Moreover, parent-focused training and workshops have been shown to positively impact reading frequency and quality. For instance, Reese et al. demonstrated that when low-income parents received training in elaborative book reading, their children made greater narrative and vocabulary gains over time (Reese et al., 2010). These findings support the need for community-based initiatives that equip parents with skills to scaffold their children’s literacy development effectively.

The critical role of community support for families facing vulnerability is well-documented, with comprehensive resources and wraparound services often necessary to support children’s development and learning effectively (Dowdall et al., 2019; Finck and Shea, 2015). Findings from the current study demonstrate that integrated community hubs can serve as a valuable source of such support. Building on previous research, the accessible location of the hub likely contributed to families’ participation and engagement, offering a convenient and welcoming space to implement early literacy strategies (Canfield et al., 2020).

Importantly, the community setting also promoted broader exposure to shared reading experiences for all children at the hub, not just those directly involved in the project. The group learning format—facilitated by familiar and trusted hub staff—appeared to enhance caregivers’ confidence in adopting the literacy strategies introduced (Smith et al., 2023). This aligns with Dowdall et al. (2019), who emphasize the added value of social support gained through group-based interventions. The hub environment also yielded benefits for staff and community partners involved in the project. For example, community partners noted that listening to families helped them better understand service needs. The process of co-design was particularly impactful, fostering creativity and motivation among staff and partners as they collaborated in shaping and delivering the project. Moreover, participants gained a deeper understanding of the value of co-design in tailoring interventions to reflect community needs and family preferences (Smith et al., 2023). Steen et al. highlight that although the advantages of co-design are sometimes overlooked, they should be recognized as a significant factor in the success of innovative, service-improving initiatives (Steen et al., 2011).

### Creating positive reading experiences

Creating positive reading experiences is central to fostering sustained engagement in shared book reading between parents and children. This theme encompasses a range of emotional, behavioral, and relational dynamics that transform reading from a routine task into a meaningful, enjoyable, and developmentally beneficial activity.

A fundamental component of positive reading experiences lies in encouraging reading for pleasure rather than framing it solely as an educational obligation. When parents approached shared reading with the primary goal of enjoyment, interactions were more relaxed, spontaneous, and emotionally rich. This approach aligns with previous researches who found that reading for pleasure is strongly associated with academic success, improved emotional wellbeing, and greater motivation for lifelong learning (Vogrinčič Čepič et al., 2024; Clark and Rumbold, 2006). Considering that Preece and Levy found that children’s enjoyment of shared book reading was a key motivator for parents to engage in shared book reading, it is suggested that having this positive experience of reading at the hub may have important implications for parents continuing to read and engage in enjoyable shared book reading interactions with their children themselves (Preece and Levy, 2020). When reading is enjoyable, it is more likely to become embedded in daily family routines and perceived as a desirable, voluntary activity by both parent and child.

Closely linked to this is fostering children’s active engagement during reading sessions. Children’s autonomy—reflected in their ability to choose books, flip through pages, ask questions, or respond to images and text—enhanced their interest and participation. This finding resonates with the principles of dialogic reading, where the child is positioned as an active conversational partner, leading to improved vocabulary, comprehension, and expressive language skills (Whitehurst et al., 1988). When children were given space to interact with the book physically and intellectually, they were more attentive and responsive, which in turn increased parents’ enjoyment and motivation to continue shared reading practices.

Another facilitator of meaningful reading experiences was the planning and structuring of reading times, particularly as part of evening or bedtime routines. Establishing predictable, dedicated times for reading created a sense of stability and emotional safety for both parent and child. Bedtime stories were valued as moments of calmness and connection, providing opportunities to wind down from the day and share closeness. Bus et al. emphasize that structured, ritualistic reading practices are associated with increased narrative skills and deeper parent–child attachment (Bus et al., 1995). These routines helped families maintain consistency, even in the face of busy schedules or fatigue, and were often described as “special times” by participating parents.

A further dimension of positive reading experiences involved reproducing positive childhood reading practices. Many parents recalled being read to as children and identified those memories as sources of comfort, inspiration, and cultural continuity. These intergenerational practices served as a blueprint for their current parenting behaviors. As noted by Sénéchal and Young, parents who experienced frequent early reading are significantly more likely to engage in similar literacy activities with their own children (Sénéchal and Young, 2008). Recreating the warmth and affection associated with their childhood reading experiences gave parents a sense of purpose and continuity, reinforcing shared book reading as both a personal and family tradition.

Finally, perhaps the most emotionally resonant aspect of creating positive reading experiences was the strengthening of parent–child bonding and intimacy. Reading together fostered close physical contact (e.g., sitting on laps, cuddling), focused attention, and opportunities for shared emotions and expressions. These interactions contributed not only to cognitive and linguistic development but also to emotional security and attachment. As described by Baker, shared reading can serve as a bonding ritual that nurtures the parent–child relationship, especially when other forms of interaction are limited due to time constraints or life stressors (Baker, 2003). Parents in the reviewed studies often described these moments as deeply meaningful, reinforcing their motivation to continue reading even when external challenges were present.

### Book and content quality

The quality of books and their content emerged as a significant factor influencing both the frequency and effectiveness of shared book reading. Parents across the studies emphasized that the characteristics of the books themselves—such as language level, rhythm and rhyme, visual appeal, and structure—played a critical role in capturing children’s interest and sustaining meaningful interaction.

Parents showed a strong preference for books that matched their children’s developmental stage—those that introduced new vocabulary without being too complex. Books that struck a balance between challenge and accessibility were seen as crucial for fostering early language development. Research by Justice et al., underscores this finding, showing that books with rich but manageable vocabulary contribute significantly to children’s expressive language skills when read interactively (Justice et al., 2005). Similarly, Snow and Goldfield noted that young children benefit most from books that offer slight linguistic stretch while still aligning with their existing language abilities (Snow and Goldfield, 1983).

Books that incorporated rhyme, rhythm, and repetition were especially engaging for children and often described by parents as “fun,” “musical,” and “easy to follow.” These elements encourage children to anticipate words, participate verbally, and develop phonological awareness—an essential precursor to reading acquisition. According to Goswami and Bryant, rhyming texts support the development of phonemic sensitivity, particularly in preschool-aged children (Goswami and Bryant, 1990).

Books with vivid illustrations, clear storylines, and age-appropriate text length were more likely to sustain children’s attention and facilitate comprehension. Visually engaging books also helped children make connections between images and narrative, reinforcing emergent literacy concepts such as print awareness and narrative sequencing. According to Donovan, illustrations not only support comprehension but also evoke emotional responses that deepen engagement with the text (Donovan, 2001).

### Child development

Most parents in this study reported that they consider the purpose of reading when choosing books to read with their children, aiming to support their child’s development (Deitcher et al., 2019). A common goal among parents was to convey a moral lesson or message through shared reading, which influenced their book choices. For example, parents who prioritized moral teaching were more inclined to choose *Where the Wild Things Are* due to its clear moral undertones (Owens, 1992).

About one-third of parents viewed narrative books as a means to address their child’s social–emotional development. They connected with the emotions portrayed in the stories and illustrations and often selected books that mirrored their child’s personal experiences or emotional states. These findings align with previous research indicating that shared reading offers a valuable context for discussing social–emotional topics, which in turn supports the development of children’s social cognition and empathy (Drummond et al., 2014; Kucirkova and Tompkins, 2014; Shapira et al., 2015). Books rich in social–emotional content tend to foster deeper conversations between parents and children about emotions and mental states (Adrian et al., 2005).

Although many parents already choose books based on their child’s emotional needs, enhancing their awareness of how mental-state themes in books can influence social and emotional development may further strengthen children’s prosocial behaviors and understanding.

### Parental motivation

A unique and significant facilitator identified was the intrinsic motivation of parents, driven by a sense of responsibility and emotional connection. Parents were not only motivated by the potential academic benefits for their children but also by the enjoyment of spending quality time together. This emotional reciprocity reinforced the practice of shared reading as both a bonding and developmental activity. Research by Baker et al. shows that parents who view reading as a pleasurable activity rather than an educational obligation are more likely to sustain the habit long-term (Baker, 2003).

Children’s visible enjoyment—expressed through laughter, anticipation, or discussion—further reinforced parental motivation. This finding underscores the importance of affective feedback loops in promoting sustained behaviors. In turn, when parents saw their children thriving emotionally and linguistically, it validated their role and deepened their commitment to the practice (Preece and Levy, 2020).

Despite the well-documented benefits of shared book reading for children’s language and literacy development, several barriers hinder its consistent practice across diverse populations. These challenges can be categorized into four main themes: limited resources and access, sociocultural and parental barriers, knowledge and skill deficits, and child engagement and communication challenges.

### Limited resources and access

Access to reading materials continues to pose a substantial barrier to shared book reading, particularly in economically disadvantaged households. This aligns with prior research linking fewer books in the home with reduced odds to initiate shared reading during infancy and less frequent reading overall (Sinclair et al., 2018; Berkule et al., 2007). Rather than focusing solely on community hubs or homes, efforts to promote literacy must adopt a dual approach that enhances access across both settings. Limited availability of books—whether due to cost, lack of local libraries, or logistical barriers—has been linked to lower initiation and frequency of shared reading in early childhood. The concept of “book deserts” captures this inequity, with research showing alarming disparities; in some low-income urban areas, there is reportedly only one age-appropriate book for every 300 children (Neuman and Moland, 2019). Addressing these gaps requires more than increasing book quantity—it involves offering diverse genres (e.g., picture books, culturally relevant stories, nonfiction, bilingual texts) and formats (e.g., print, digital, audio) that appeal to different family needs and contexts. Digital interventions, such as tablet-based libraries, have shown promise in reaching underserved populations and expanding access to early literacy materials (Kalil et al., 2025). Programs like Reach Out and Read and Dolly Parton’s Imagination Library also illustrate the impact of combining book distribution with trusted community touchpoints (Crosh et al., 2024). Future strategies must ensure that both physical and digital resources are developmentally appropriate, engaging, and readily accessible, especially for families navigating socioeconomic adversity (Vouyoukas et al., 2025).

### Sociocultural and parental barriers

Parental beliefs and cultural practices significantly influence shared reading behaviors. Some parents perceive reading as an activity reserved for formal education settings, leading to delayed introduction of shared reading at home. Additionally, parents who were not read to during their own childhood may lack models for engaging in shared reading with their children. A study by Levy et al. found that parents’ personal reading histories and beliefs about reading purposes affect their shared reading practices (Levy et al., 2018).

Time constraints and parental fatigue also pose significant barriers. Working parents, especially those juggling multiple jobs or caregiving responsibilities, often report insufficient time or energy to engage in regular shared reading sessions. Furthermore, parental mental health issues, such as depression or stress, can diminish the quality and frequency of reading interactions, as emotional availability is crucial for meaningful engagement (Swain et al., 2017).

### Knowledge and skill deficits

A lack of awareness and confidence in selecting appropriate storybooks and applying effective reading strategies continues to hinder parental engagement in shared reading. Many caregivers are unsure how to choose books that align with their child’s developmental stage, language ability, or interests. This uncertainty can lead to the use of materials that are either too advanced or insufficiently stimulating, reducing the quality and impact of the reading experience. Similarly, unfamiliarity with interactive techniques—such as asking open-ended questions, prompting predictions, or encouraging child-led discussions—can limit the developmental benefits of shared reading. Esmaeeli and Wagner emphasize that parental knowledge is a key factor in establishing rich home literacy environments (Esmaeeli and Wagner, 2022).

To address this, both parents and community-hub stakeholders—such as librarians, early educators, and program facilitators—require structured guidance on selecting developmentally appropriate storybooks. Practical resources such as age-banded book lists, bilingual recommendations, and culturally relevant titles can help families identify books that are engaging and suitable for their child’s level. Additionally, training stakeholders to support parents in this selection process can increase confidence, reduce anxiety, and promote consistent reading routines. Language barriers and low parental literacy further complicate this issue, particularly in linguistically diverse communities where reading materials in native languages are limited (Brown et al., 2019). As such, literacy initiatives must prioritize inclusive and accessible strategies that empower families to make informed choices and engage more effectively in shared reading practices.

### Child engagement and communication challenges

Parents often encounter challenges when engaging young children—particularly infants and toddlers—who may display limited attention spans or seem disinterested in reading activities. These behaviors can lead caregivers to assume their child is not developmentally ready for shared reading. However, evidence consistently shows that early exposure to books, even before full comprehension develops, plays a foundational role in language acquisition and the establishment of reading routines (Crosh et al., 2024).

The emergence of digital books has introduced both new possibilities and important considerations for shared reading. On one hand, e-books can enhance engagement through multimedia features such as audio narration, animation, and word highlighting—especially helpful in maintaining the attention of easily distracted children. This can be particularly useful in households with limited access to physical books or for children with specific learning needs. On the other hand, several studies caution that these digital features may unintentionally undermine the quality of parent–child interaction, which is essential for language-rich exchanges during reading (Willoughby et al., 2015). For example, Strouse and Ganea (2017) found that while e-books can support certain learning outcomes, they are also associated with fewer verbal interactions and reduced conversational turn-taking compared to print books. These disruptions may negatively affect the dialogic quality of shared reading and, consequently, literacy development.

The findings from the reviewed studies underscore the importance of balancing the benefits of digital accessibility with the developmental strengths of print-based reading. Parents and early literacy programs should be equipped with guidance on selecting high-quality digital books that encourage interaction rather than passive viewing. At the same time, print books remain indispensable, especially in promoting physical closeness, emotional bonding, and sustained joint attention—all key factors for fostering emergent literacy. As such, future literacy efforts should adopt a complementary approach, helping families integrate both formats based on their child’s age, attention span, and learning context while preserving the interactive nature of shared reading.

### Strengths and limitations of study

This meta-synthesis applied a rigorous and transparent methodology, following ENTREQ guidelines and using critical realism as a theoretical framework. A broad search across multiple databases and a structured quality assessment with the CASP checklist enhanced credibility. The inclusion of studies from diverse countries and participant backgrounds enriched the findings, while the use of thematic synthesis and MAXQDA software strengthened systematic analysis. The study also offers practical insights that can inform interventions and future research in promoting parent–child shared book reading.

This meta-synthesis also has some limitations. One notable limitation of this meta-synthesis is the relatively small number of studies included in the final analysis (*N* = 9). While each study provided rich qualitative data and contributed meaningfully to the synthesis, the limited scope may constrain the generalizability of the findings, particularly across diverse cultural, linguistic, and socioeconomic contexts. Most of the included studies were conducted in high-income, English-speaking countries, which may not fully capture the range of shared reading experiences globally. Cultural and structural differences—such as access to books, parental literacy, and norms around reading—may influence shared reading practices differently. It would be better to include a broader sample of studies, particularly from underrepresented regions and non-English sources, and consider incorporating high-quality gray literature to mitigate potential publication bias. Although the inclusion criteria did not restrict based on child developmental status, none of the included studies explicitly focused on shared book reading experiences involving children with disabilities. Therefore, the findings of this synthesis primarily reflect the experiences of families with typically developing children, and further research is needed to explore facilitators and barriers in populations with additional developmental needs. Finally, differences in study designs and analytic approaches may have introduced variability into the synthesis.

### Research and clinical implications

The findings of this meta-synthesis underscore the importance of developing community-based interventions that promote shared book reading as both a literacy-building and relationship-enhancing practice. Clinically, early childhood and family health professionals—such as pediatric nurses, community health workers, and early intervention specialists—should be encouraged to embed literacy promotion into routine practice by offering guidance on developmentally appropriate book selection, shared reading techniques, and culturally responsive materials. Community hubs, libraries, and early childhood centers can serve as critical access points for distributing books, delivering parent workshops, and modeling interactive reading strategies, particularly in underserved communities.

From a research standpoint, future studies should explore how shared book reading practices can be adapted to low-income and culturally diverse populations. There is a need to examine scalable interventions—both print and digital—that are effective in resource-limited settings. In particular, longitudinal and implementation studies could help evaluate the long-term impact of community-embedded literacy programs on language, social–emotional development, and parent–child bonding. Additionally, involving caregivers in the co-design of interventions may improve their relevance, accessibility, and uptake. Expanding research beyond high-income settings will also support the development of inclusive frameworks that ensure equitable access to early literacy opportunities across socio-economic and cultural boundaries.

## Conclusion

This meta-synthesis provides a comprehensive understanding of the factors that facilitate and challenge parent–child shared book reading practices. The findings highlight that access to books, parental support and education, the creation of positive reading experiences, and parents’ intrinsic motivation play crucial roles in promoting shared reading. At the same time, barriers such as limited resources, sociocultural beliefs, time constraints, parental mental health, and knowledge gaps must be addressed to enhance engagement in shared reading activities.

The study underscores the importance of community-based interventions, culturally responsive resources, and parental education programs in supporting early literacy development. Future efforts should focus on expanding access to books, offering practical support for parents, and promoting shared reading as an enjoyable and meaningful family activity across diverse social and cultural contexts. By addressing both facilitators and challenges, initiatives can be better designed to strengthen early literacy foundations and foster lasting parent–child connections through shared book reading.

## Footnotes

^1^https://casp-uk.net/wp-content/uploads/2018/03/CASP-Qualitative-Checklist-2018_fillable_form.pdf
